# Vitamin C intake and cognitive function in older U.S. adults: nonlinear dose–response associations and effect modification by smoking status

**DOI:** 10.3389/fnut.2025.1585863

**Published:** 2025-06-04

**Authors:** Xingchen He, Yijia Lin, Xinyi Wu, Min Li, Tianyu Zhong, Yanhong Zhang, Xuliang Weng

**Affiliations:** ^1^The Affiliated Guangzhou Hospital of TCM of Guangzhou University of Chinese Medicine, Guangzhou, China; ^2^Sleep Research Institute of Traditional Chinese Medicine, Guangzhou Medical University, Guangzhou, China

**Keywords:** vitamin C, cognitive function, NHANES, older adults, dose–response relationship, smoking status

## Abstract

**Objective:**

To investigate the association between vitamin C intake and cognitive function in U.S. older adults, focusing on dose–response characteristics and effect modification of key subgroups.

**Methods:**

Utilizing data from the 2011–2014 National Health and Nutrition Examination Survey (NHANES), this cross-sectional study included 2,801 adults aged ≥ 60 years. Total vitamin C intake was assessed via standardized 24-h dietary recalls and supplement questionnaires. Cognitive function was evaluated using the Consortium to Establish a Registry for Alzheimer’s Disease (CERAD) Word Learning Test, Animal Fluency Test (AFT), and Digit Symbol Substitution Test (DSST). Multivariate adjusted linear regression models, smooth curve fitting, and stratified regression analyses were employed to examine associations and effect modification.

**Results:**

Our analysis revealed a nonlinear dose–response relationship between vitamin C intake and cognitive performance. In fully adjusted models, participants in the highest intake quartile (Q4) showed significantly better performance on the Auditory Fluency Test (AFT; *β* = 1.11, 95% CI: 0.37–1.85) and the Digit Symbol Substitution Test (DSST; *β* = 3.35, 95% CI: 1.49–5.21) compared to those in the lowest quartile (Q1). Threshold analyses indicated that cognitive protection for DSST peaked at an intake of 500 mg/day, while AFT benefits plateaued at 120 mg/day. Stratified analyses further demonstrated that the cognitive benefits of vitamin C were more pronounced among smokers (DSST: *β* = 0.59 per 100 mg/day, *p* = 0.0009), with no significant associations observed in non-smokers.

**Conclusion:**

Vitamin C intake is associated with improved cognitive function in older U.S. adults, with distinct dose-dependent and domain-specific threshold effects. Smoking status significantly modifies this relationship, suggesting that personalized supplementation strategies targeting smokers may enhance cognitive protection.

## 1 Introduction

The increasing global population aging has made cognitive decline a significant challenge in geriatric health management. According to World Health Organization (WHO) estimates, about 5–8% of individuals aged 60 years or older worldwide exhibit varying degrees of cognitive impairment (1). The socioeconomic burden of neurodegenerative disorders, particularly Alzheimer’s disease, exceeded US$1 trillion in 2020 (2). This underscores the importance of identifying modifiable dietary factors that can influence cognitive trajectories. In this context, vitamin C (ascorbic acid) has emerged as a plausible candidate due to its dual role as a water-soluble antioxidant and neuromodulator. Mechanistically, vitamin C exerts neuroprotective effects through free radical scavenging, reducing oxidative stress, and participating in the synthesis of dopamine and norepinephrine. Experimental models also reveal its ability to regulate blood–brain barrier permeability and reduce *β*-amyloid deposition, a key pathological feature of Alzheimer’s disease (3, 4). Moreover, emerging evidence suggests that nicotine, a key psychoactive component in tobacco, may exert cognitive-enhancing effects through activation of neuronal nicotinic acetylcholine receptors (nAChRs), which are widely distributed in brain regions associated with memory and attention, including the prefrontal cortex and hippocampus (5, 6). NAChRs can modulate neurotransmitter release and synaptic plasticity, further implicating lifestyle factors like smoking in cognitive health (7).

However, epidemiological evidence remains inconsistent. For example, prospective cohort studies like the Rotterdam Study have shown positive correlations between plasma vitamin C levels and cognitive performance (8, 9), while no such associations were found in cross-sectional analyses such as the National Health and Nutrition Examination Survey (NHANES). Notably, there are still research gaps regarding dose–response relationships and population-specific effects in U.S. older adults. This gap is addressed in the current study through the innovative use of NHANES data from 2011 to 2014.

Leveraging NHANES’s standardized 24-h dietary recalls and validated cognitive modules (e.g., CERAD Word Learning Test) (10, 11), this study pioneers three critical inquiries: (1) Whether vitamin C intake is independently associated with cognitive function in U.S. older adults; (2) Whether a nonlinear dose–response relationship exists between vitamin C intake and cognitive impairment; and (3) Effect modification within key subgroups (e.g., diabetes status, smoking status). These findings will provide evidence-based insights for developing targeted dietary intervention strategies.

## 2 Materials and methods

### 2.1 Study design and data source

This cross-sectional investigation utilized data from the 2011–2014 National Health and Nutrition Examination Survey (NHANES), a nationally representative surveillance program employing multistage stratified probability sampling to capture the non-institutionalized civilian population in the United States. Sociodemographic variables (age, gender, household income, educational attainment) and health-related lifestyle factors (alcohol consumption, smoking status, physical activity levels) were collected through structured interviews using validated questionnaires (12). The National Center for Health Statistics (NCHS) Research Ethics Review Board approved all study protocols, with written informed consent obtained from all participants prior to data collection (13). To ensure methodological rigor, we adhered to STROBE (Strengthening the Reporting of Observational Studies in Epidemiology) guidelines and implemented stringent exclusion criteria final analytical cohort comprised 2,801 adults aged ≥60 years (57).

### 2.2 Vitamin C intake

The NHANES study utilized 24-h dietary recalls and supplement questionnaires to comprehensively assess vitamin C intake. Participants completed two interviews: an in-person session at Mobile Examination Centers (MEC) and a follow-up telephone interview within 3–10 days. Supplement users reported product names, doses, and frequencies, with vitamin C-specific data extracted for analysis. Ion protocols to account for bioavailability variations. Nutrient intake calculations integrated averaged values from both dietary recalls and supplement logs, employing standardized conversion protocols. This dual-phase design minimized recall bias while capturing day-to-day dietary fluctuations, ensuring robust estimation of total vitamin C exposure.

### 2.3 Cognitive function assessment

The NHANES investigation employed a comprehensive cognitive assessment battery to evaluate memory retention and executive functioning among participants. A pivotal component of this evaluation was the Consortium to Establish a Registry for Alzheimer’s Disease (CERAD) Word List Learning Test, which systematically measures the capacity for acquiring new verbal information (14, 15). During this standardized protocol, participants were instructed to audibly recite a list of 10 semantically unrelated nouns, followed by three consecutive trials of immediate free recall, with each trial scored on a 0–10 scale. To assess delayed memory consolidation, a parallel recall test was administered approximately 8–10 min after the initial learning phase, utilizing the same scoring metric. Complementing this evaluation, the Animal Fluency Test (AFT) served as a dual-domain probe of linguistic proficiency and executive control by requiring participants to generate as many animal names as possible within a 60-s interval, with each valid response awarded one point (16, 17). Concurrently, processing speed and cognitive flexibility were quantified through the Digit Symbol Substitution Test (DSST), a time-constrained neuropsychological instrument (18, 19). In this paradigm, participants matched numerical digits (0–9) to corresponding geometric symbols using a reference key within a two-minute time frame, completing up to 133 paired associations. Performance was scored based on accurately transcribed symbol-digit pairs, with possible scores ranging from 0 to 133. Across all assessments, higher composite scores consistently correlated with superior cognitive performance, reflecting enhanced memory encoding efficiency, lexical retrieval capacity, and psychomotor processing speed. The tripartite testing framework provided multidimensional insights into age-related cognitive trajectories while maintaining ecological validity through standardized administration protocols (20, 21).

### 2.4 Covariates

The selection of covariates was rigorously informed by existing epidemiological literature, encompassing demographic, anthropometric, and behavioral determinants: sex, chronological age, racial/ethnic identity, body mass index (BMI), marital status, educational attainment, smoking behavior, alcohol consumption patterns, and diabetes comorbidity. Age stratification followed a tripartite division (60–69, 70–79, and ≥ 80 years) to capture differential aging trajectories. Racial/ethnic categorization comprised non-Hispanic White, non-Hispanic Black, Mexican American, and Other Race groups, reflecting U.S. census classifications. Marital status was dichotomized into partnered (married/cohabitating) and unpartnered states. BMI categorization adhered to WHO standards: normal weight (< 25 kg/m^2^), overweight (25–30 kg/m^2^), and obese (≥ 30 kg^2^) (22). Educational attainment was operationalized as <9 years (primary education), 9–12 years (secondary education), and >12 years (tertiary education). Smoking status differentiated never-smokers (<100 lifetime cigarettes) from ever-smokers (≥100 cigarettes). Alcohol consumption was quantified through 12-month recall as light/non-drinking (≤1 drink/day), moderate (2–3 drinks/day), and heavy (≥3 drinks/day), with standard drink equivalents calibrated to USDA guidelines.

### 2.5 Statistical analysis

Baseline characteristics of study participants were compared using chi-square tests for categorical variables and analysis of variance (ANOVA) for continuous measures. Vitamin C intake was stratified into quartiles, with the lowest quartile (Q1) designated as the reference category. Multivariate adjusted linear regression models were sequentially constructed to assess the associations between vitamin C intake and three cognitive function scores, quantified by *β* coefficients with 95% confidence intervals (CIs). Model 1 represented the crude association without adjustment; Model 2 adjusted for demographic covariates (age, sex, and race/ethnicity); Model 3 further incorporated clinical and behavioral confounders (BMI, smoking status, alcohol consumption patterns, and diabetes status). A linear trend test was performed by treating quartile categories as ordinal variables. To explore potential nonlinear relationships, dose–response curves between vitamin C intake and cognitive dysfunction were modeled using smooth curve fitting, optimized through sensitivity analyses. Subgroup analyses employed stratified regression frameworks, where continuous covariates were categorized based on clinical thresholds or population-derived quartiles. Interaction terms were introduced to evaluate heterogeneity in vitamin C effects across subgroups defined by biological sex, diabetes status, and smoking history. All regression models applied Bonferroni correction for the three primary cognitive outcomes (CERAD, AFT, DSST), adjusting the significance threshold to *α* = 0.017 (0.05/3). Subgroup analyses were interpreted cautiously as exploratory, with interaction terms evaluated at α = 0.05. All statistical procedures were implemented in R version 4.1.1 (R Foundation for Statistical Computing) with supplementary validation via EmpowerStats 2.0 software.

## 3 Results

**Figure 1:**
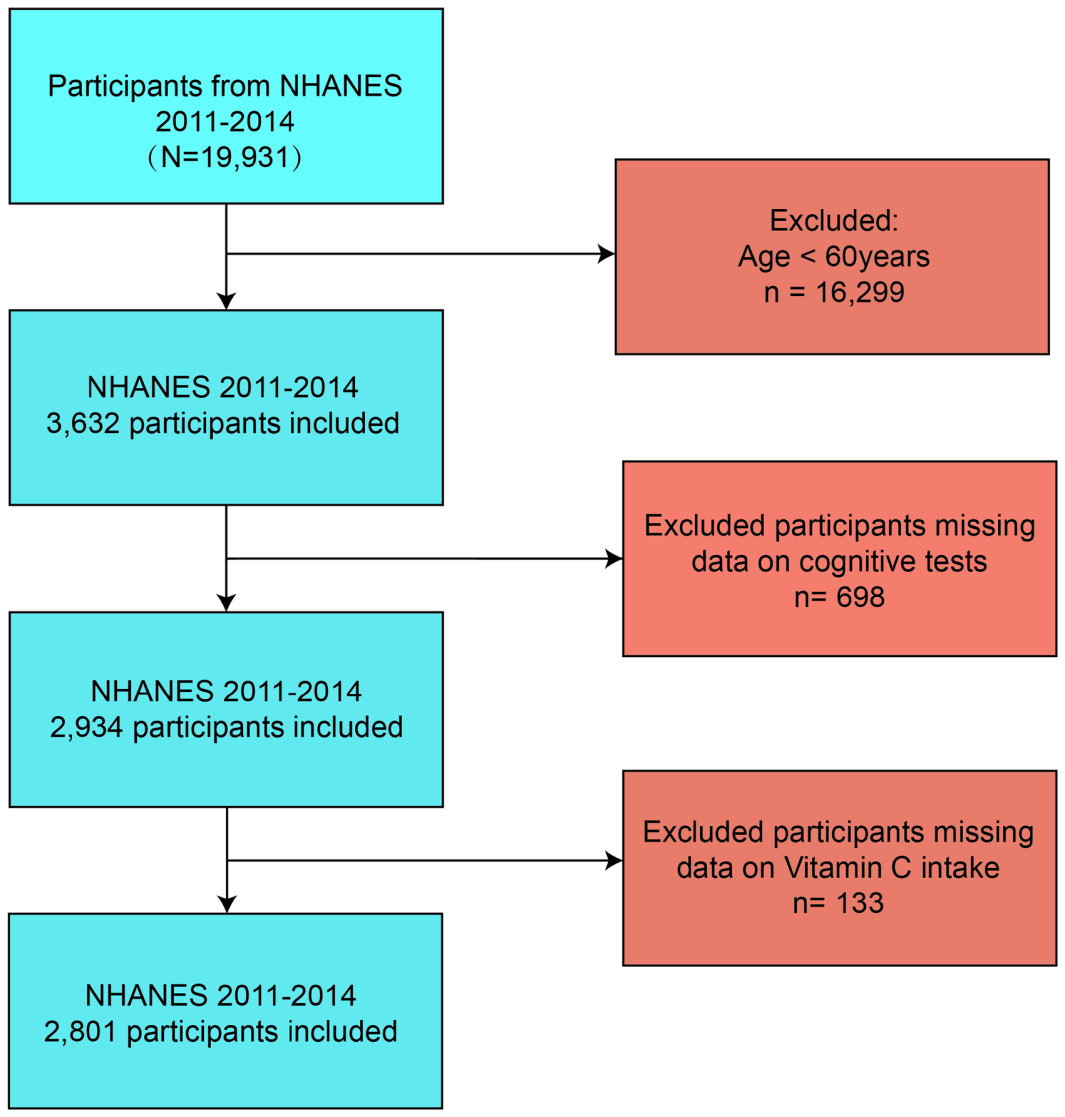
Flow diagram of the screening and enrollment of study participants.

In the 2011–2014 cycle, the NHANES included 19,931 participants. Exclusion criteria comprised individuals aged < 60 years (*n* = 16,299), those missing dietary vitamin C data (*n* = 133), and participants lacking cognitive assessments (*n* = 698). Consequently, the final analytical sample consisted of 2,801 participants. Figure 1 details the selection process.

### 3.1 Participants’ characteristics at baseline

Table 1 delineates the baseline characteristics of 2,801 U.S. adults aged ≥ 60 years (49.1% male, 48.2% female) stratified by vitamin C intake quartiles. The cohort demonstrated a mean age of 69.41 ± 6.76 years, with median daily vitamin C intake at 98.6 mg (mean: 186 mg). Notably, 45% of participants fell below Dietary Reference Intakes (DRI) recommendations (90 mg/d for males, 75 mg/d for females). Cognitive performance metrics revealed median scores of 25 (CERAD immediate recall), 46 (DSST processing speed), and 16 (AFT verbal fluency), with elevated quartiles of vitamin C intake exhibiting superior cognitive outcomes (CERAD: 25.32 ± 6.55; DSST: 49.47 ± 16.26; AFT: 17.33 ± 5.39). Mild cognitive impairment was operationalized as scoring within the lowest cognitive function quartile (Q1) across composite measures.

**Table 1:** Baseline characteristics of the included population.

Characteristic	*N*	Q1 (≦42.8)	Q2 (42.9–98.45)	Q3 (98.55–190)	Q4 (190.3–4,226)	*p*-value
Total	2,801	*N* = 700	*N* = 699	*N* = 701	*N* = 701	
Age (years)	69.41 ± 6.76	68.67 ± 6.54	68.67 ± 6.69	69.76 ± 6.82	70.54 ± 6.81	<0.001
60–70	1,528 (54.55%)	421 (60.143%)	413 (59.084%)	360 (51.355%)	334 (47.646%)	
70–80	823 (29.38%)	191 (27.286%)	189 (27.039%)	218 (31.098%)	225 (32.097%)	
>80	450 (16.07%)	88 (12.571%)	97 (13.877%)	123 (17.546%)	142 (20.257%)	
Gender, *n* (%)						0.061
Male	1,374 (49.05%)	350 (50.00%)	361 (51.65%)	314 (44.79%)	349 (49.79%)	
Female	1,427 (50.95%)	350 (50.00%)	338 (48.35%)	387 (55.21%)	352 (50.21%)	
Race, *n* (%)						<0.001
Mexican American	239 (8.53%)	66 (9.43%)	72 (10.30%)	67 (9.56%)	34 (4.85%)	
Other Hispanic	286 (10.21%)	79 (11.29%)	79 (11.30%)	72 (10.27%)	56 (7.99%)	
Non-Hispanic White	1,350 (48.20%)	292 (41.71%)	306 (43.78%)	355 (50.64%)	397 (56.63%)	
Non-Hispanic Black	667 (23.81%)	194 (27.71%)	189 (27.04%)	148 (21.11%)	136 (19.40%)	
Other	259 (9.25%)	69 (9.86%)	53 (7.58%)	59 (8.42%)	78 (11.13%)	
Education level (year), *n* (%)						<0.001
<9	312 (11.14%)	111 (15.86%)	91 (13.02%)	60 (8.56%)	47 (6.70%)	
9–12	391 (13.96%)	128 (18.29%)	104 (14.88%)	91 (12.98%)	68 (9.70%)	
>12	2,098 (74.90%)	496 (65.56%)	504 (72.1%)	549 (78.32%)	586 (83.6%)	
Marital status, *n* (%)						0.025
Married or living with a partner	1,625 (58.05%)	385 (55%)	392 (56.08%)	414 (59.06%)	425 (62.05%)	
Living alone	1,175 (41.95%)	315 (45%)	307 (43.92%)	287 (40.94%)	276 (37.95%)	
Smoke, *n* (%)						<0.001
Yes	1,422 (50.77%)	384 (54.86%)	374 (53.51%)	351 (50.07%)	313 (44.65%)	
No	1,379 (49.23%)	316 (45.14%)	325 (46.49%)	350 (49.93%)	388 (55.35%)	
Alcoholic (Avg drinks), *n* (%)						0.001
1–2		194 (54.80%)	221 (54.84%)	245 (61.56%)	247 (60.40%)	
2–3		128 (36.16%)	154 (38.21%)	129 (32.41%)	154 (37.65%)	
>3		32 (9.04%)	28 (6.95%)	24 (6.03%)	8 (1.96%)	
BMI (kg/m^2^)						0.014
	29.08 ± 6.36	29.53 ± 7.00	29.32 ± 6.22	28.99 ± 6.00	28.48 ± 6.13	
Diabetes, *n* (%)						0.003
Yes	656 (23.42%)	186 (26.57%)	187 (26.75%)	160 (22.82%)	123 (17.55%)	
No	2,145 (76.58%)	484 (69.14%)	476 (68.10%)	510 (72.75%)	545 (77.75%)	
CERAD, Mean ± SD						0.005
	24.86 ± 6.52	24.16 ± 6.51	24.83 ± 6.57	25.14 ± 6.40	25.32 ± 6.55	
DSST, Mean ± SD						<0.001
	45.99 ± 17.23	42.65 ± 17.28	44.80 ± 17.86	47.03 ± 16.78	49.47 ± 16.26	
AFT, Mean ± SD						<0.001
	16.62 ± 5.49	15.76 ± 5.37	16.34 ± 5.48	17.05 ± 5.57	17.33 ± 5.39	

Vitamin C intake quartile thresholds were defined as Q1 (≤ 42.8 mg/d), Q2 (42.9–98.45 mg/d), Q3 (98.55–190.3 mg/d), and Q4 (190.3–4,226 mg/d). Demographically, non-Hispanic White predominance intensified across ascending quartiles (*p* < 0.01), paralleled by a dose-dependent gradient in age (68.67 ± 6.54 to 70.54 ± 6.81 years), educational attainment (> 12 years: 65.56 to 83.6%), and partnered marital status (55 to 62.05%). Behavioral analyses uncovered inverse correlations between vitamin C intake and current smoking prevalence (54.86% in Q1 vs. 44.65% in Q4, *p* < 0.001), though alcohol consumption demonstrated a U-shaped pattern (54.80% light drinkers in Q1 vs. 60.39% in Q4, *p* = 0.001). Clinically, higher intake quartiles correlated with progressive BMI reduction (29.53 ± 7.00 to 28.48 ± 6.13 kg/m^2^, *p* = 0.01) and declining diabetes prevalence (26.57 to 17.55%, *p* = 0.03). These gradients persisted after age-sex standardization, suggesting potential nutrient-behavior synergies (23).

### 3.2 Multivariate adjusted linear regression model

Table 2 details the association of the Vitamin intake with Cognitive function. Utilizing nationally representative NHANES 2011–2014 data, we constructed multivariable-adjusted linear regression models to investigate the dose–response relationship between quartile-stratified vitamin C intake (Q1–Q4, with Q1 as reference) and cognitive performance metrics (CERAD immediate recall, AFT verbal fluency, DSST processing speed). The analytical framework employed progressive covariate adjustment: Model 1 (crude association), Model 2 (demographic-adjusted: age, sex, race/ethnicity), and Model 3 (fully-adjusted: marital status, educational attainment, BMI, smoking behavior, alcohol consumption patterns, and diabetes comorbidity). Quartile thresholds were established through equidistant categorization of vitamin C intake levels, ensuring uniform exposure intervals across groups.

**Table 2:** Association of the vitamin intake with cognitive function.

Variables / Categories	Model 1 *β* (95%CI)*, p-*value	Model 2 *β* (95%CI)*, p-*value	Model 3 *β* (95%CI)*, p-*value
CERAD score			
Vitamin intake			
Q1	Reference	Reference	Reference
Q2	0.671 (−0.011, 1.353) 0.0538	0.707 (0.079, 1.335) 0.02735	−0.041 (−0.863, 0.780) 0.92146
Q3	0.984 (0.303, 1.665) 0.00468	1.061 (0.431, 1.690) < 0.001	0.256 (−0.578, 1.089) 0.54778
Q4	1.160 (0.478, 1.841) < 0.001	1.387 (0.754, 2.020) < 0.001	0.336 (−0.503, 1.174) 0.43286
*P* for trend	0.0032	<0.001	0.9941
AFT score			
Vitamin intake			
Q1	Reference	Reference	Reference
Q2	0.585 (0.013, 1.156) 0.04500	0.491 (−0.037, 1.019) 0.06837	0.253 (−0.475, 0.981) 0.49583
Q3	1.291 (0.720, 1.862) < 0.001	1.264 (0.734, 1.794) < 0.001	1.292 (0.554, 2.030) 0.0062
Q4	1.575 (1.004, 2.146) < 0.001	1.570 (1.038, 2.103) < 0.001	1.108 (0.365, 1.850) 0.00352
*P* for trend	<0.001	<0.001	0.005
DSST score			
Vitamin intake			
Q1	Reference	Reference	Reference
Q2	2.150 (0.362, 3.937) 0.01848	2.185 (0.673, 3.697) 0.00466	1.120 (−0.699, 2.940) 0.22773
Q3	4.386 (2.600, 6.172) < 0.001	4.170 (2.654, 5.686) < 0.001	2.093 (0.248, 3.938) 0.02632
Q4	6.821 (5.035, 8.607) < 0.001	6.374 (4.850, 7.898) < 0.001	3.351 (1.494, 5.208) 0.00042
*P* for trend	<0.001	<0.001	<0.001

A robust positive trend emerged across all models (*P*-trend < 0.05), with cognitive score increments persisting after sequential adjustment for potential confounders. In the fully adjusted Model 3, participants in upper intake quartiles demonstrated clinically meaningful cognitive advantages: Q4 groups exhibited > 1.0 standard deviation unit improvements in AFT (Q4 *β* = 1.11, 95%CI 0.37–1.85) and DSST (Q4 *β* = 3.35, 95%CI 1.49–5.21) scores compared to Q1, whereas Q2 showed non-significant associations (AFT *β* = 0.25, 95%CI −0.48–0.98; DSST *β* = 1.12, 95%CI −0.70–2.94). Sensitivity analyses confirmed model stability through variance inflation factor diagnostics and residual normality assessments. The threshold effect observed between Q2 and Q3 suggests potential existence of a biological intake plateau for cognitive benefits, possibly corresponding to the Dietary Reference Intake threshold (75–90 mg/d). These findings align with neuroprotective mechanisms involving vitamin C’s antioxidant capacity and its role in dopamine neurotransmission modulation (24).

### 3.3 Smooth curve fitting and threshold effect analysis

Operationalizing cognitive dysfunction through quartile-based categorization of composite cognitive scores, we defined participants in the lowest quartile (Q1) as the cognitive impairment group. Generalized additive models (GAMs) revealed significant nonlinear inverse associations between vitamin C intake and cognitive impairment risk for both the DSST and AFT. Segmented regression models validated threshold effects at distinct intake levels: For DSST performance, a critical inflection point emerged at 500 mg/d (log-likelihood ratio test *p* = 0.006), with each 10 mg/d increment below this threshold conferring a 3% reduction in cognitive impairment risk (OR = 0.97, 95%CI 0.96–0.99). Beyond 500 mg/d, no significant association was observed (OR = 1.01 per 10 mg/d, 95%CI 0.99–1.02).

**Figure 2:**
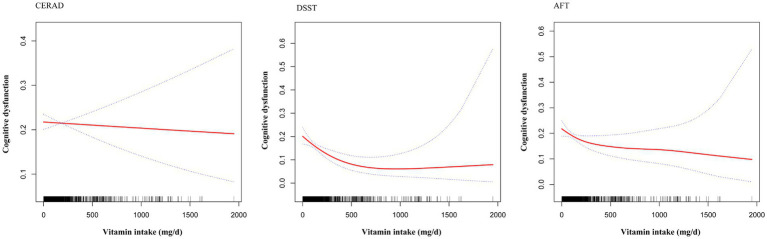
The dose–response relationship between vitamin C intake and cognitive impairment.

The AFT analysis identified a lower threshold at 120 mg/d (log-likelihood ratio test *p* = 0.029), where pre-threshold intake increments demonstrated stronger protective effects (4% risk reduction per 10 mg/d: OR = 0.96, 95%CI 0.92–0.90), plateauing post-threshold (OR = 1.00, 95%CI 0.99–1.00). Notably, the DSST’s higher threshold (500 mg/d) potentially reflects differential neurobiological demands for processing speed versus verbal fluency, possibly related to vitamin C’s varied roles in prefrontal cortex versus hippocampal metabolism. In contrast, the Consortium to Establish a Registry for Alzheimer’s Disease (CERAD) test exhibited a linear protective gradient across the full intake spectrum (*P*-linear < 0.001), suggesting domain-specific mechanisms in episodic memory preservation. Figure 2 illustrates the dose–response relationship between vitamin C intake and cognitive impairment among older adults in the United States. Table 3 illustrates the threshold effect analysis.

**Table 3:** Threshold effect analysis.

Variables / Categories	Adjusted OR (95%CI)	*p*-value
DSST		
Vitamin C intake (mg)		
<500	0.97 (0.96, 0.99)	0.0003
≥500	1.01 (0.99, 1.02)	0.3696
log-likelihood ratio test	0.006	
AFT		
Vitamin C intake (mg)		
<120	0.96 (0.92, 0.99)	0.0110
≥120	1.00 (0.99, 1.00)	0.3225
log-likelihood ratio test	0.029	

### 3.4 Subgroup analyses and sensitivity analysis

**Figure 3:**
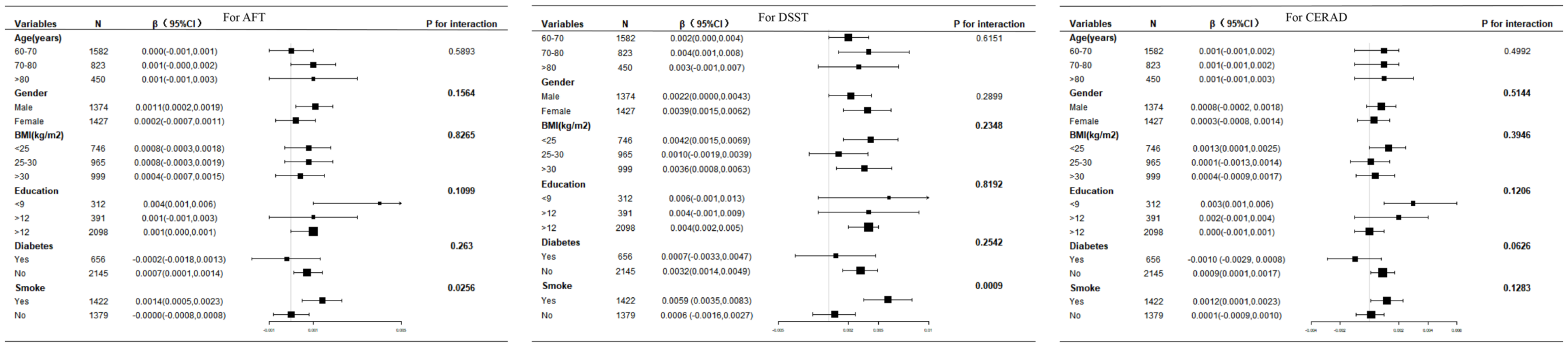
The results of the subgroup analyses.

Figure 3 presents the results of the subgroup analyses evaluating potential effect modifications across biological and sociodemographic subgroups, including gender (male vs. female), age categories (60–70, 70–80, >80 years), BMI classifications (<25, 25–30, ≥ 30 kg/m^2^), educational attainment (<9, 9–12, >12 years), diabetes status (yes/no), and smoking history (current smokers vs. non-smokers). Notably, smoking status emerged as a statistically significant modifier of the vitamin C-cognition association (DSST: *P*-interaction = 0.0009; AFT: *P*-interaction = 0.0256). Among smokers, each 100 mg/d increase in vitamin C intake was associated with improved DSST performance (*β* = 0.59, 95% CI: 0.35–0.83), whereas no significant association was observed in non-smokers (*β* = 0.02, 95% CI -0.16–0.27). Similar trends were noted for AFT scores (smokers: *β* = 0.14, 95% CI: 0.05–0.23; non-smokers: *β* = −0.00, 95% CI: −0.08–0.08). To further investigate potential benefits of supplement use, we conducted a sensitivity analysis comparing cognitive function between participants with vitamin C intake > 500 mg/day (presumed to represent supplement users) and those with intake ≤ 500 mg/day (Supplementary Table 1). The analysis revealed no significant association between high-dose vitamin C (> 500 mg/day, presumably from supplements) and cognitive function improvement (DSST OR = 1.01, 95% CI: 0.99–1.02; AFT OR = 1.00, 95% CI: 0.98–1.01; CERAD OR = 0.99, 95% CI: 0.98–1.00). Importantly, no significant interaction effects were detected in other subgroups (all *P*-interaction > 0.05), reinforcing smoking status as the primary modifier in the observed diet-cognition relationship. These findings highlight the necessity of considering smoking behavior in nutritional interventions targeting cognitive health.

## 4 Discussion

Our study provides novel evidence on the dose-dependent association between vitamin C intake and cognitive performance in older U.S. adults, complementing the neuroprotective effects of multivitamin supplementation observed in the COSMOS trials. While COSMOS emphasized synergistic actions of micronutrients (25), our findings suggest vitamin C may exert independent neuroprotection via oxidative stress modulation, potentially serving as a key active component in multivitamin regimens (26).

The observed threshold for cognitive performance highlights a potential upper limit of vitamin C’s neuroprotective efficacy. Notably, while supplementation enables individuals to achieve high intake levels (e.g., Q4 range up to 4,226 mg/d), our data conclusively demonstrate no additional cognitive benefits beyond 500 mg/day. This plateau may reflect saturation of vitamin C’s biological mechanisms—such as SVCT2 transporter capacity or antioxidant recycling pathways—which limit further systemic or neural uptake at excessive doses (27–29). Importantly, these findings caution against indiscriminate high-dose supplementation, as benefits plateau while potential risks (e.g., oxalate nephropathy, iron overload) may increase (30, 31). Interestingly, this saturation phenomenon contrasts with findings from the COSMOS study, where multivitamins containing 500 mg of vitamin C demonstrated broader cognitive benefits (22), indicating that single-nutrient interventions may be subject to inherent efficacy limitations. The differential thresholds for various cognitive domains—lower for semantic memory and executive function (AFT: 120 mg/day) compared to higher for processing speed (DSST: 500 mg/day)—reflect the multi-tiered mechanisms of vitamin C: dopaminergic modulation in frontal circuits may be responsive to lower doses, whereas neurovascular enhancement required for complex integration tasks necessitates higher intakes. This is consistent with Alzheimer’s disease models that show preferential protection of the hippocampus, advocating for domain-specific nutritional strategies (32, 33). The linear association observed with the CERAD test may indicate sustained dose-responsive benefits for declarative memory or reflect limited. Statistical power to detect subtle thresholds, thereby highlighting the need for larger cohorts integrated with biomarker assessments.

The pronounced cognitive benefits of vitamin C observed in smokers may reflect a synergistic interaction between nicotine’s transient activation of nAChR-mediated neuroprotection and vitamin C’s multimodal actions, including antioxidant defense and catecholamine biosynthesis cofactor functions (34–36). Experimental studies indicate that nicotine activates pro-survival pathways (e.g., PI3K/AKT) and enhances synaptic plasticity via nAChRs (37, 38), which may transiently improve processing speed and executive function. However, chronic smoking simultaneously exacerbates oxidative stress, depleting endogenous antioxidants such as vitamin C (39). Smokers typically require approximately 2 times the vitamin C intake of non-smokers to achieve adequate serum vitamin C concentrations (40). In this context, vitamin C supplementation may not only mitigate oxidative damage but also potentiate nicotine-induced neuroprotection by preserving redox balance. This explains why smoking status emerged as a critical effect modifier, likely through interacting metabolic pathways: chronic smoke exposure depletes plasma vitamin C creating an antioxidant deficit where supplemental intake gains amplified neuroprotection (41). Nicotine-induced blood–brain barrier permeability (42) may enhance vitamin C delivery to prefrontal (AFT-associated) and parietal-cerebellar networks (DSST-associated). The 4-fold stronger DSST response versus AFT might reflect processing speed’s dependence on myelination and synaptic plasticity—microstructures vulnerable to smoking-related neuroinflammation (43). Conversely, the lack of significant association between vitamin C intake and cognitive function in non-smoking populations may be related to the “antioxidant threshold effect.” When baseline oxidative stress levels are low, the neuroprotective effects of additional vitamin C intake might fail to exceed the detection sensitivity of cognitive assessment tools (44). Public health implications are twofold: smokers may require more to target executive dysfunction, while non-smokers might need combinatorial approaches with vitamin E or flavonoids to bypass antioxidant plateaus (45, 46).

Vitamin C exerts neuroprotective effects via multiple mechanisms. As the principal water-soluble antioxidant, it scavenges free radicals within the central nervous system and inhibits *β*-amyloid aggregation (47, 48). Vitamin C also promotes collagen synthesis, thereby maintaining the structural integrity of cerebral blood vessels (49). Moreover, it modulates the activity of dopamine-*β*-hydroxylase, which in turn affects executive functions mediated by the prefrontal cortex (50). However, Vitamin C’s role in cognitive function may extend beyond antioxidant activity. As a cofactor for dopamine-*β*-hydroxylase, vitamin C is essential for norepinephrine synthesis in the prefrontal cortex (51, 52). This mechanism could directly enhance executive function and processing speed, particularly in smokers who exhibit catecholamine dysregulation due to nicotine exposure. Additionally, vitamin C supports collagen synthesis, maintaining cerebrovascular integrity and cerebral blood flow—critical for age-related cognitive preservation (53, 54). Collectively, these mechanisms constitute a multifaceted defense network against age-related cognitive decline. In the present study, only 45% of elderly participants met the Dietary Reference Intake (DRI) standards for vitamin C, suggesting an inadequate vitamin C nutritional status among older adults in the United States. Although we have observed an association between vitamin C intake and cognitive function, the specific numerical value for the daily recommended intake should be approached with caution. Additional oxidative stress, such as that induced by smoking, may influence daily requirements, while adverse health conditions may deplete plasma and body stores of vitamin C. Therefore, we suggest that future research further investigate the body status and plasma saturation of vitamin C to determine a more accurate daily recommended intake (26, 55, 56).

This study has several methodological limitations. First, residual confounding may persist due to unaccounted dietary covariates that interact with vitamin C metabolism. Second, the cross-sectional design precludes causal inference, as temporal ambiguity raises concerns about reverse causation—particularly given that cognitive decline may influence dietary habits. Third, while NHANES protocols ensure standardized quantification of total vitamin C intake (dietary + supplemental), the absence of plasma ascorbate measurements prevents direct evaluation of systemic bioavailability differences between these two sources. Future directions should integrate prospective designs, RCTs, and plasma biomarkers to establish causality while exploring gene-nutrient interactions (e.g., SVCT2 polymorphisms) that may personalize dosing strategies. This work advances nutritional neuroscience by delineating vitamin C’s non-linear, domain-specific cognitive benefits and identifying smoking status as a key modifier—insights critical for precision nutrition in aging populations.

## 5 Conclusion

This study highlights the association between vitamin C intake and cognitive function and explores the dose-dependent relationship between vitamin C intake and cognitive impairment in older Americans with neuroprotective thresholds of 500 mg/day for processing speed (DSST) and 120 mg/day for verbal fluency (AFT). Smoking status significantly altered these effects, with smokers experiencing greater cognitive benefits, possibly due to oxidative stress alleviation. These findings advocate for targeted interventions—dietary enrichment or supplementation—to address age-related cognitive decline, especially in at-risk populations. Due to cross-sectional limitations, longitudinal validation and exploration of gene-nutrient interactions are necessary to develop precise nutritional strategies.

## Data Availability

The original contributions presented in the study are included in the article/Supplementary material, further inquiries can be directed to the corresponding authors.
