# Supplementary material for: Vitamin C intake and cognitive function in older U.S. adults: nonlinear dose–response associations and effect modification by smoking status
Source: Front Nutr. 2025 Jun 4;12:1585863. doi: 10.3389/fnut.2025.1585863 (PMC12173858; doi:10.3389/fnut.2025.1585863)
Supplement: Supplementary file 1 [file Table_1.docx]

| **The sources of vitamin C** | **N** | **Cognitive dysfunction** | | |
| --- | --- | --- | --- | --- |
|  |  | DSST OR (95%)CI | AFT OR (95%)CI | CERAD OR (95%)CI |
| Dietary sources of vitamin C (Vitamin C ≤ 500 mg/d) | 2536 | 0.97 (0.96,0.99) | 0.99 (0.98, 1.00) | 1.00 (0.99, 1.02) |
| Vitamin C supplements  (Vitamin C > 500 mg/d) | 265 | 1.01 (0.99,1.02) | 1.00 (0.98, 1.01) | 0.99 (0.98, 1.00) |
